# Critical role of surface chemical modifications induced by length shortening on multi-walled carbon nanotubes-induced toxicity

**DOI:** 10.1186/1743-8977-9-46

**Published:** 2012-11-27

**Authors:** Cyrill Bussy, Mathieu Pinault, Julien Cambedouzou, Marion Julie Landry, Pascale Jegou, Martine Mayne-L'hermite, Pascale Launois, Jorge Boczkowski, Sophie Lanone

**Affiliations:** 1 Inserm U955, Equipe 04, Créteil, F-94000, France; 2 Faculté de Médecine, Université Paris-Est, UMR 955, Créteil, F-94000, France; 3 Laboratoire de Physique des Solides, UMR CNRS 8502, Université Paris-Sud 11, Orsay, cedex F-91405, France; 4 CEA, IRAMIS, SPAM, Laboratoire Francis Perrin (CEA-CNRS URA 2453), Gif-sur-Yvette, 91191, France; 5 DSM/IRAMIS/SPCSI/LCSI, CEA-Saclay, Gif-sur-Yvette, Cedex 91191, France; 6 AP-HP, Hôpital Henri Mondor, Service de Physiologie Explorations Fonctionnelles, Créteil, 94000, France; 7 Centre Hospitalier Intercommunal, Service de pneumologie et pathologie professionnelle, Créteil, 94010, France; 8 Faculté de Médecine, 8, rue du Général Sarrail, Créteil, 94000, France; 9 Current address: Nanomedicine laboratory, Centre for Drug Delivery Research, UCL School of Pharmacy, University College London, London, WC1N 1AX, UK; 10 Current address: Institut de Chimie Séparative de Marcoule, UMR 5257 CEA/CNRS/UMII/ENSCM, Centre de Marcoule, BP 17171, Bagnols sur Cèze, Cedex F-30207, France

**Keywords:** Carbon nanotubes, Macrophages, Length, Surface chemistry

## Abstract

Given the increasing use of carbon nanotubes (CNT) in composite materials and their possible expansion to new areas such as nanomedicine which will both lead to higher human exposure, a better understanding of their potential to cause adverse effects on human health is needed. Like other nanomaterials, the biological reactivity and toxicity of CNT were shown to depend on various physicochemical characteristics, and length has been suggested to play a critical role. We therefore designed a comprehensive study that aimed at comparing the effects on murine macrophages of two samples of multi-walled CNT (MWCNT) specifically synthesized following a similar production process (aerosol-assisted CVD), and used a soft ultrasonic treatment in water to modify the length of one of them. We showed that modification of the length of MWCNT leads, unavoidably, to accompanying structural (i.e. defects) and chemical (i.e. oxidation) modifications that affect both surface and residual catalyst iron nanoparticle content of CNT. The biological response of murine macrophages to the two different MWCNT samples was evaluated in terms of cell viability, pro-inflammatory cytokines secretion and oxidative stress. We showed that structural defects and oxidation both induced by the length reduction process are at least as responsible as the length reduction itself for the enhanced pro-inflammatory and pro-oxidative response observed with short (oxidized) compared to long (pristine) MWCNT. In conclusion, our results stress that surface properties should be considered, alongside the length, as essential parameters in CNT-induced inflammation, especially when dealing with a safe design of CNT, for application in nanomedicine for example.

## Background

Potential adverse effects of carbon nanotubes (CNT) on human health are of great concern, especially if we consider their increasing use in composite materials [1] and also their exploration as innovative solutions for biomedical applications [1-5]. Like other nanomaterials, the biological reactivity and toxicity of CNT were shown to depend on numerous physicochemical characteristics including length, diameter, structural defects, surface area, tendency to agglomerate, dispersibility in solution, presence and nature of catalyst residues, as well as surface chemistry [6-20].

Among those features, the length has been suggested to play a critical role in the CNT biological reactivity after inhalation. According to a well-established paradigm for high aspect ratio nanomaterials, CNT with length superior to that of phagocytic cells can induce an inflammatory response, which is an important event contributing to tissue remodeling and carcinogenesis. In a seminal study, Poland and coworkers [21] showed that ‘long’ multi-walled CNT (MWCNT) -the term ‘long’ meaning that a significant proportion of them was longer than 15 μm- induced acute and chronic peritoneal inflammation and also the formation of granulomas on the mesothelial lining in mice, while shorter MWCNT (with no reliable count obtained for CNT with a length > 15 μm) did not. The same group demonstrated that CNT length is also an important determinant of their retention in the pleural space and of their subsequent effects in terms of inflammation and fibrosis development in mice [22]. However, a major drawback of such studies [21-23] was the use of different suppliers to provide the various MWCNT. Due to discrepancies in production methods, the CNT were therefore differing not only in length but also in many other physicochemical characteristics. Indeed, the authors reported larger diameters for longer CNT, and different contents in soluble metals between the different CNT studied were also described [21,23]. As mentioned before, these physicochemical differences could in turn affect the CNT biological reactivity and subsequent toxicity, and thus should be considered alongside the variation in length to assess the toxicological profile of CNT.

On the basis of the length paradigm and the hypothesis that length is not the only parameter to consider when evaluating the cytotoxic effects of CNT, we designed a comprehensive *in vitro* study that aimed at comparing the biological effects, on murine macrophages, of two samples of MWCNT which differed in length but were of similar diameter and residual catalyst metal content. Both samples were specifically produced for our study following a similar synthesis process (i.e. aerosol-assisted CCVD Catalytic Chemical Vapor Deposition). Materials of the batch referred to as “short” (S-CNT) were obtained by reducing the length of pristine MWCNT (initially grown aligned as in a carpet for 10 min, and referred to as PS-CNT, where ‘P’ stands for ‘Precursor’) using “long lasting” (i.e. 7 weeks) soft ultrasonic treatment in water [24]. The batch referred to as “long MWCNT” (L-CNT) were pristine CNT that were grown aligned for 2 minutes without further treatment [25]. Along with length, other physicochemical features were extensively characterized by several material science methods, namely electron microscopies (transmission - TEM, and scanning - SEM), thermogravimetric analysis (TGA), X-ray diffraction (XRD) and X-ray photo-electron Spectroscopy (XPS), so as to evaluate in depth the physico-chemical differences between the two samples. We showed that modification of the length of MWCNT leads unavoidably to additional structural (i.e. defects) and chemical (i.e. oxidation) modifications that affect both CNT surface and residual catalyst iron nanoparticles. The biological response of murine macrophages to the two different MWCNT studied was then evaluated in terms of cell viability, pro-inflammatory potential and oxidative stress. Unexpectedly, we observed an enhanced pro-inflammatory and pro-oxidative response only with the short (oxidized) MWCNT, compared to the long (pristine) MWCNT, which can be also attributed to structural defects and surface oxidation -both introduced during the shortening process- rather than to the length reduction only.

## Results

### CNT characterization

**Figure 1:**
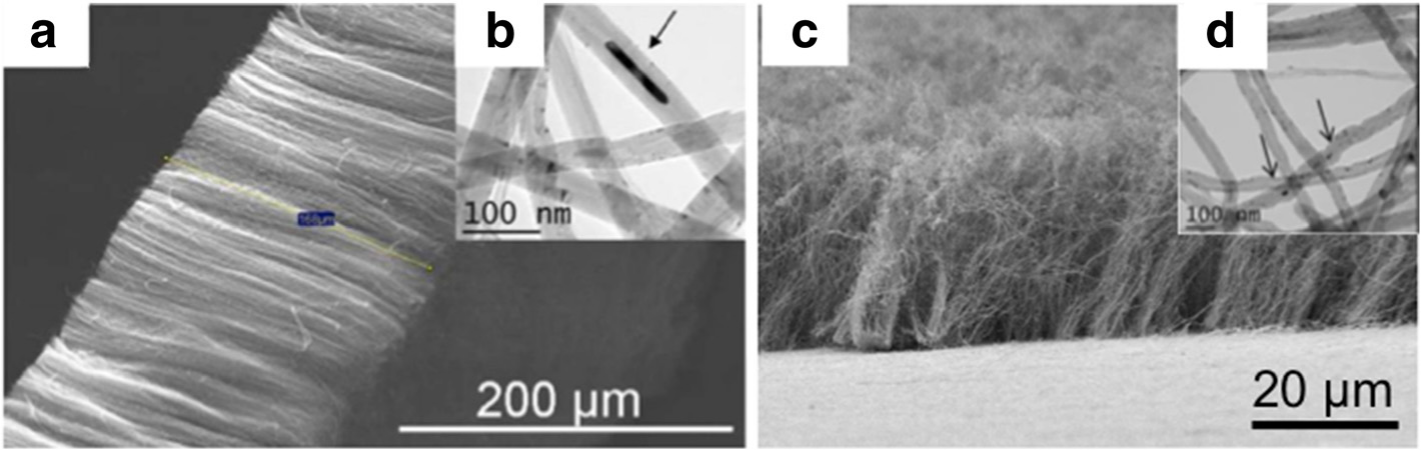
**Electron microscopy images of PS- and L-CNT.** Scanning electron microscopy (SEM, panel **a**, and **c**) images and Transmission electron microscopy (TEM, panel **b**, and **d**) images of PS-CNT (before length reduction by ultrasonic treatment, panel a and b) and L-CNT (panel c-d). Black arrows point toward iron-based nanoparticles.

**Figure 2:**
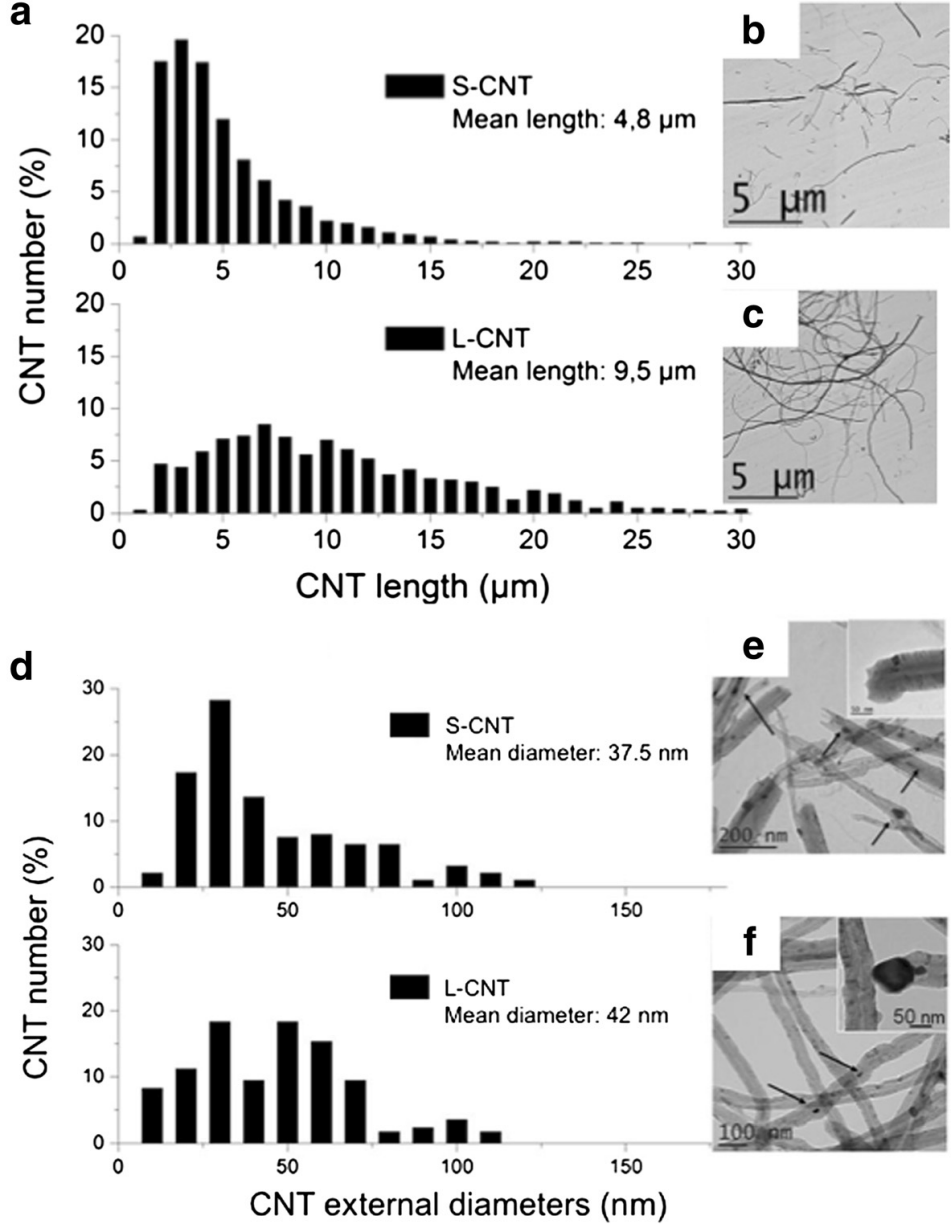
**Measurement of CNT length and external diameter.** Determination of length (panel **a**) and external diameter (panel **d**) distributions of S- and L-CNT. TEM images of S-CNT (panel **b** and **e**) and L-CNT (panel **c** and **f**). Black arrows point toward iron-based nanoparticles.

Following their synthesis, samples of PS-CNT and L-CNT, both in the form of aligned CNT carpets covering the reactor walls, were collected by scratching off the reactor walls. Typical Scanning and Transmission Electron Microscopy (SEM and TEM) images of PS-CNT carpets (Figure 1a and b) and of L-CNT (Figure 1c, d) are presented in Figure 1. After preparation of the S-CNT sample by long-term ultrasonic treatment of PS-CNT, both S-CNT and L-CNT samples were suspended in serum-free cell culture medium and the final length range distribution was measured from several TEM pictures (more than 1000 MWCNT were counted). Mean length was measured at 4.8 μm for S-CNT and 9.5 μm for L-CNT (Figure 2a, b, and c). Size distribution for S-CNT was: <5μm: 54%; <10μm: 86%; <15μm: 97%; <20μm: 99%; and for L-CNT was: <5μm: 11%; <10μm: 53%; <15μm: 79%; <20μm: 92%. Mean external diameter was 37.5 nm for S-CNT and 42 nm for L-CNT (Figure 2d, e, and f). The similarity of the two samples’ mean diameters is noteworthy because it could hardly be found in the literature with CNT provided by different suppliers [21]. TEM observations showed that both CNT samples contained almost no carbon-based by-products such as amorphous carbon, but they contained iron-based particles (i.e. catalyst particles) either attached at their basis and encapsulated in carbon sheets, or entrapped inside their hollow core [25,26] (see Figure 1b, 1d and Figure 2e, 2f). Occasionally, such iron-based nanoparticles encapsulated in carbon sheets were also detected on the surface of the CNT. The latter location was however less frequent in S-CNT sample compared to L-CNT sample.

TGA analysis showed that Fe content was about 5.8 wt.% for S-CNT and 4.8 wt.% for L-CNT.

**Figure 3:**
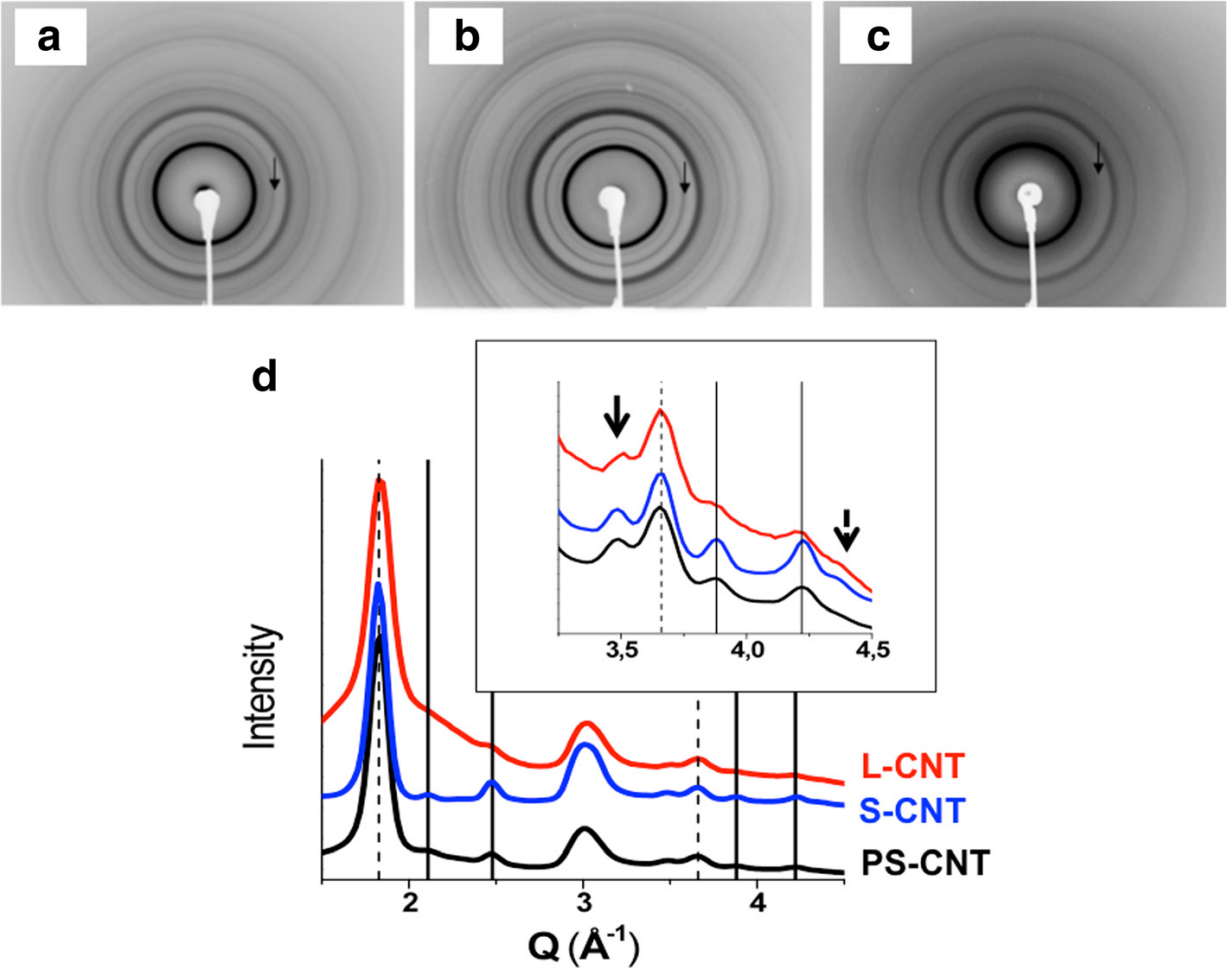
**X-ray diffraction of the different CNT.** X-ray diffraction patterns of modified CNT before (panel **a**, PS-CNT) and after length reduction (panel **b**, S-CNT), and L-CNT (panel **c**), powder-like samples being placed in capillaries. The corresponding diffraction diagrams are drawn in (**d**). The solid and dotted lines indicate positions of diffraction peaks characteristic of iron oxide Fe_3_O_4_ nanoparticles and of inter-wall distance in MWCNT, respectively. The solid and dotted arrows in the inset point towards diffraction peaks characteristic of γ and α-iron nanoparticles, while their most intense diffraction peaks are located around 3 Å^-1^, where CNT contribution is also found. The broad peak below the 002 CNT peak (around 1.83 Å^-1^) is due to scattering from the glass capillary; its relative intensity with respect to other peaks is meaningless since it only reflects the density of the powder in the capillary.

Figure 3 shows XRD patterns of the modified CNT (before (PS-CNT) and after (S-CNT) length shortening process) and of L-CNT, together with the matching diffraction diagrams. Peak indexation shows that the three samples were made of MWCNT and of γ-Fe, α-Fe and Fe_3_O_4_ nanoparticles (see refs [26-28] for details). In Figure 3d, the two diagrams for S-CNT and L-CNT were normalized to the intense 002 diffraction peak which is related to the inter-wall distance in MWCNT, located at 1.83 Å^-1^. The main difference between the S-CNT and L-CNT diagrams was the intensity of the diffraction peaks related to magnetite Fe_3_O_4_ nanoparticles. Comparison of integrated peak intensities showed that the mass content in magnetite nanoparticles normalized to the mass content in CNT was two times higher in S-CNT compared to L-CNT. Moreover, comparison between the diagrams of PS-CNT and S-CNT after the ultrasound (US) shortening process showed that there is a 2-fold increase in the iron oxide content over the 7-week-long US treatment in water. Due to the cutting/opening effect of US, a fraction of the metallic Fe material of the CNT sample may have been in direct contact with the environment (i.e. water) in which the MWCNT where suspended, leading to its further oxidation.

**Figure 4:**
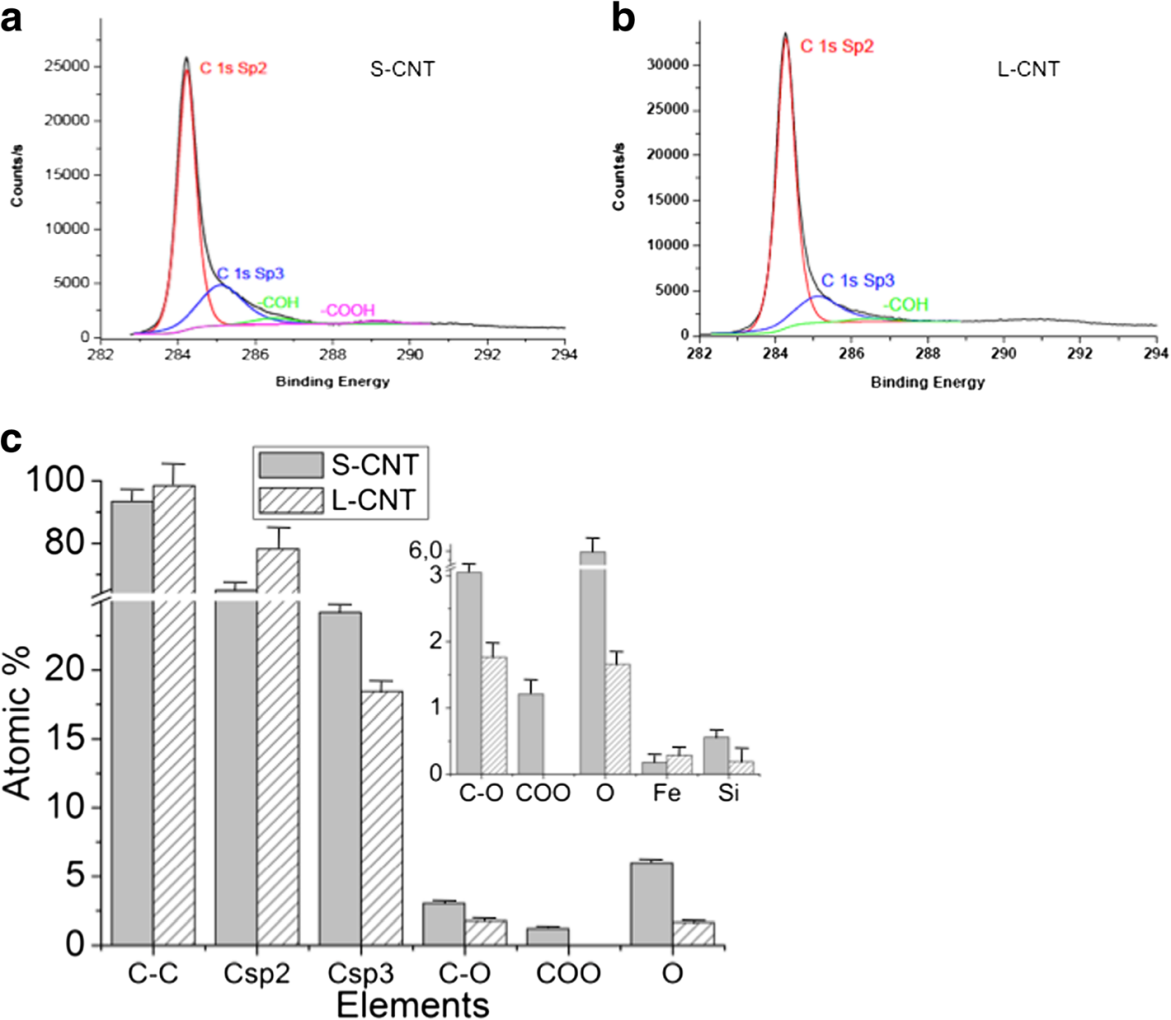
**XPS analysis of S- and L-CNT.** XPS spectra C1s core level for (**a**) S-CNT and (**b**) L-CNT and (**c**) corresponding quantitative analyses of the surface chemical composition extracted from the C1s, O1s and Fe2p spectra. Data in (**c**) are given as mean ± SEM.

X-ray induced photoelectron Spectroscopy (XPS) analysis of MWCNT surface was then performed to determine whether the surface chemistry was different between L-CNT and S-CNT samples (Figure 4, estimation of the depth analyzed is of a few nm for CNT). Figure 4a and 4b show the C1s spectrum of S- and L-CNT, which could be resolved into four characteristic peaks (see [29] for detailed XPS analysis). The binding energies of 284.4-284.7 eV, 285–285.2 eV, 286.4 eV and 289.2 eV [30] were attributed to C sp2 (C on a non-defective nanotube), C sp3 (commonly related to structural defects), C-OH and O=C-OH, respectively (Figure 4b). O1s and Fe2p core levels spectra are not displayed here but quantification of the atomic percent for O, C and Fe species is given in Figure 4c. It shows an increase of C structural defects (associated to the increase of the sp3/sp2 ratio from 0.23 to 0.37) in S-CNT compared to L-CNT, which can be attributed to the long-term US treatment of S-CNT. These results were consistent with the increase of structural defects observed by TEM at CNT tips (see e.g. the broken tube termination in the inset of Figure 2e). Moreover, the XPS analysis revealed an important evolution in the global O contribution (1.66% for L-CNT and 5.98% for S-CNT) combined with the specific presence of carboxylic functions on S-CNT surface in addition to a slight increase in hydroxyl groups (1 atomic %) which has already been observed for CNT treated by long US treatment in water [24]. These joined increases of the C sp3 and O signals revealed that dangling bonds - formed during the ultrasonic treatment process - rapidly reacted with water to give oxygen-based functionalities. Finally, the lower amount of Fe detected for S-CNT compared to L-CNT (0.1 atomic %, Figure 4c) could be explained by the loss, during the long-term US treatment process, of the Fe-based catalytic particles usually present at the CNT basis. The small amount of silicon reported in Figure 3(c) can be attributed to contaminations from the quartz reactor and the glass bottles used to disperse and store the CNT samples.

The main physicochemical characteristics of S-CNT and L-CNT, collected from complementary analysis methods presented above, are summarized in Table 1.

**Table 1:** Characteristics of the short and long CNT (S-CNT and L-CNT respectively)

	**S-CNT**	**L-CNT**
**Diameter** (nm), mean (extremes)	37.5 (10–120)	42 (10–120)
**Length**		
-Mean length (μm)	4.8	9.5
- CNT <5μm (%)	54	11
- CNT <10 μm (%)	86	53
- CNT <15 μm (%)	97	79
- CNT < 20 μm (%)	99	92
**Metal content**		
- Iron (%) (TGA)	5.8	4.8
**XPS (atomic %)**		
- sp3/sp2	0.37	0.23
- O	5.98	1.66
- Carboxylic functions	1.21	0
- OH function	3.05	1.77
**XRD**		
Sample components	MWCNT + Fe3O4, alpha iron and beta iron nanoparticles	MWCNT + Fe3O4, alpha iron and beta iron nanoparticles
Ratio of the amounts of iron oxide and CNT, normalized to that in L-CNT	2	1
**Endotoxin**	ND	ND

ND: not detectable.

### CNT internalization

**Figure 5:**
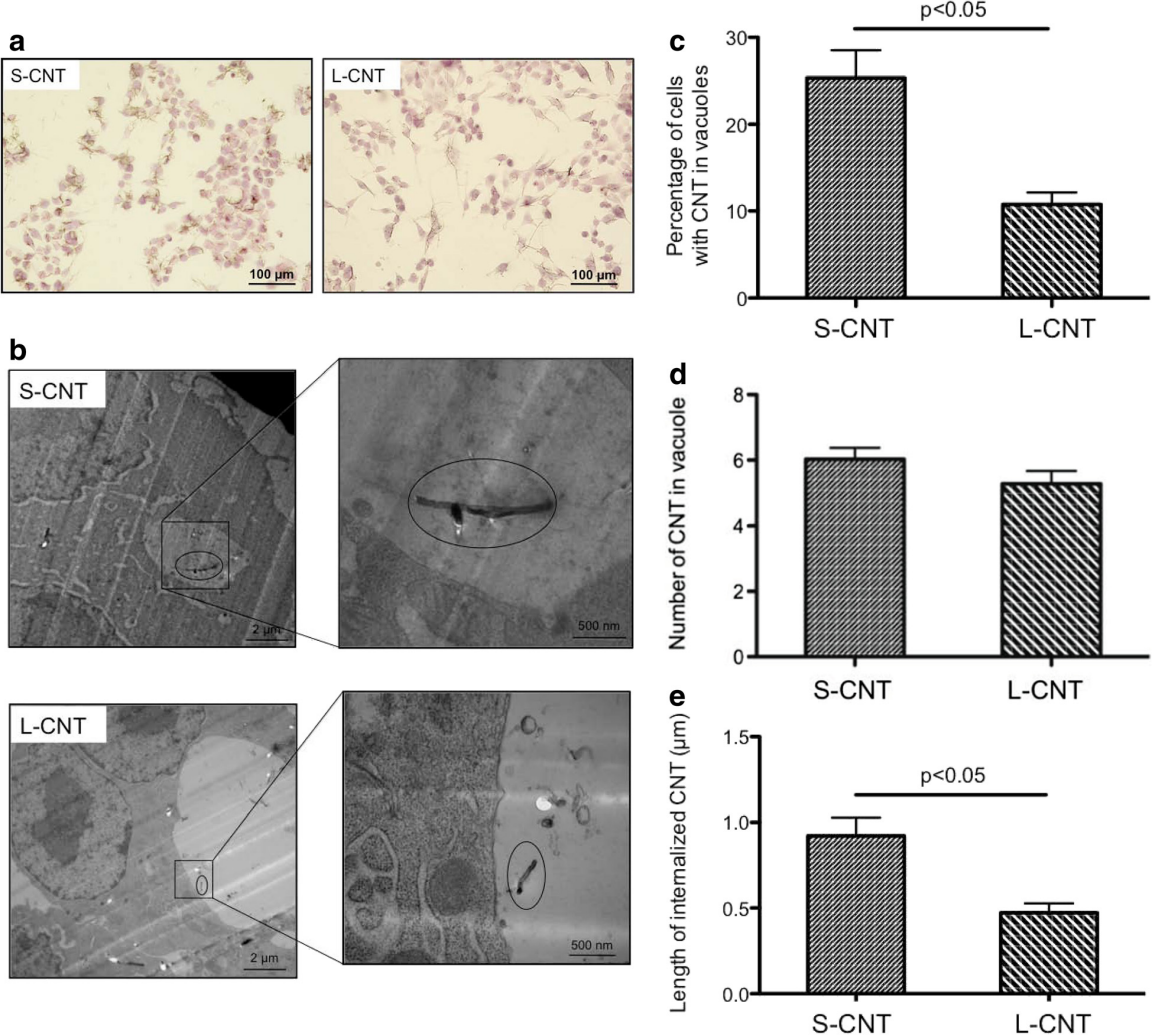
**Microscopy images of macrophages exposed to S- and L-CNT.** Optical microscopy (panel **a**) and TEM (panel **b**) images of RAW 264.7 macrophages exposed to 10 μg/mL of S- and L-CNT for 24 hours. Quantification of the percentage of cells with CNT-containing vesicles (panel **c**). Quantification of the number of CNT inside vesicles (panel **d**). Quantification of CNT length inside vesicles (panel **e**). Data are represented as mean ± SEM. P<0.05 between S- and L-CNT.

Figure 5 shows representative light microscopy (Figure 5a) and TEM (Figure 5b) images of macrophages exposed for 24 h to S- or L-CNT. Both MWCNT were internalized by macrophages, mainly within vesicles (Figure 5b) but also free in cytoplasm (Additional file 1: Figure S1). Quantitative analysis of internalization in vesicles showed that 24 h after the initial exposure the percentage of cells containing MWCNT was significantly greater with S-CNT compared to L-CNT (p <0.05, Figure 5c), while at the same time the number of CNT per vesicle was similar for both S- and L-CNT (p=0.15, Figure 5d). Moreover, the mean length of S-CNT that were internalized in cells was higher than that of L-CNT (p<0.05, Figure 5e). In both cases, it was corresponding to the fraction of CNT smaller than 5 μm (min: 0.14 μm; max: 2.65 μm for S-CNT, and min: 0.13 μm; max: 1.42 μm for L-CNT).

### Effect of CNT on cell viability

**Figure 6:**
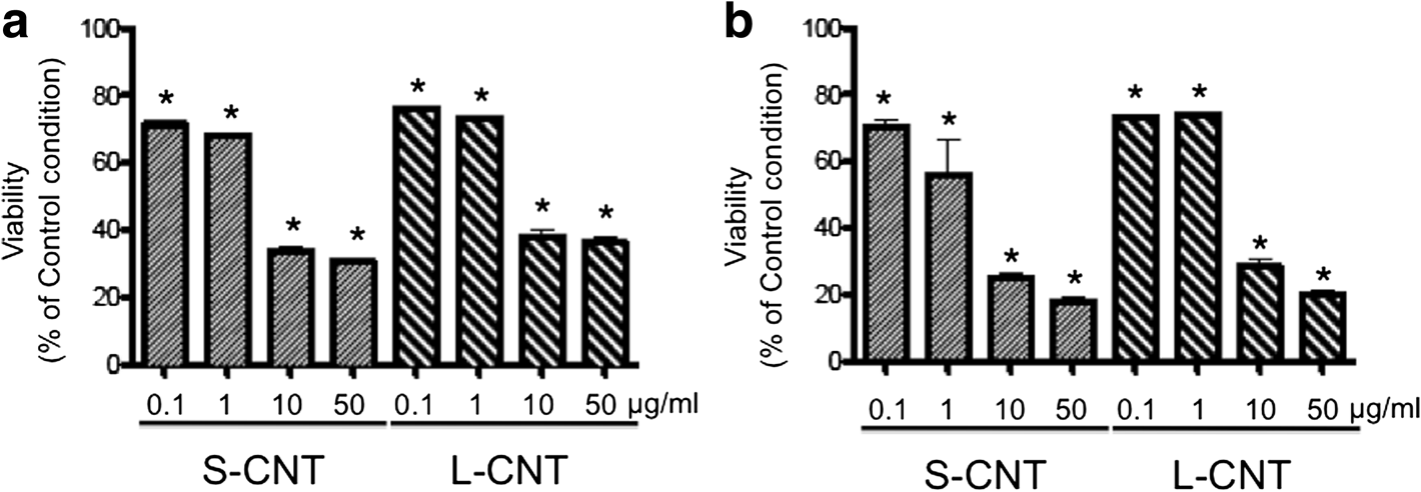
**Viability of macrophages exposed to S- and L-CNT.** Quantification of cell viability using WST-1 assay in RAW 264.7 macrophages exposed to 0.1-50 μg/mL of S- or L-CNT for 6 (panel **a**) or 24 (panel **b**) hours. *: p<0.05 versus control condition. S-CNT: short CNT. L-CNT: long CNT.

Cell viability was assessed using several tests. The WST-1 assay showed a dose-dependent decrease in mitochondrial metabolism after 6 and 24h exposures to both MWCNT (Figure 6 and Additional file 2: Figure S2). The effect of S- and L-CNT was similar at both time points and at all concentrations tested. Similar results were observed with DNA content quantification (data not shown).

### Pro-inflammatory and pro-oxidant effects of CNT

Cellular inflammatory response was analyzed by quantifying the mRNA expression and protein concentration of two pro-inflammatory cytokines, namely TNF-α and CXCL2.

**Figure 7:**
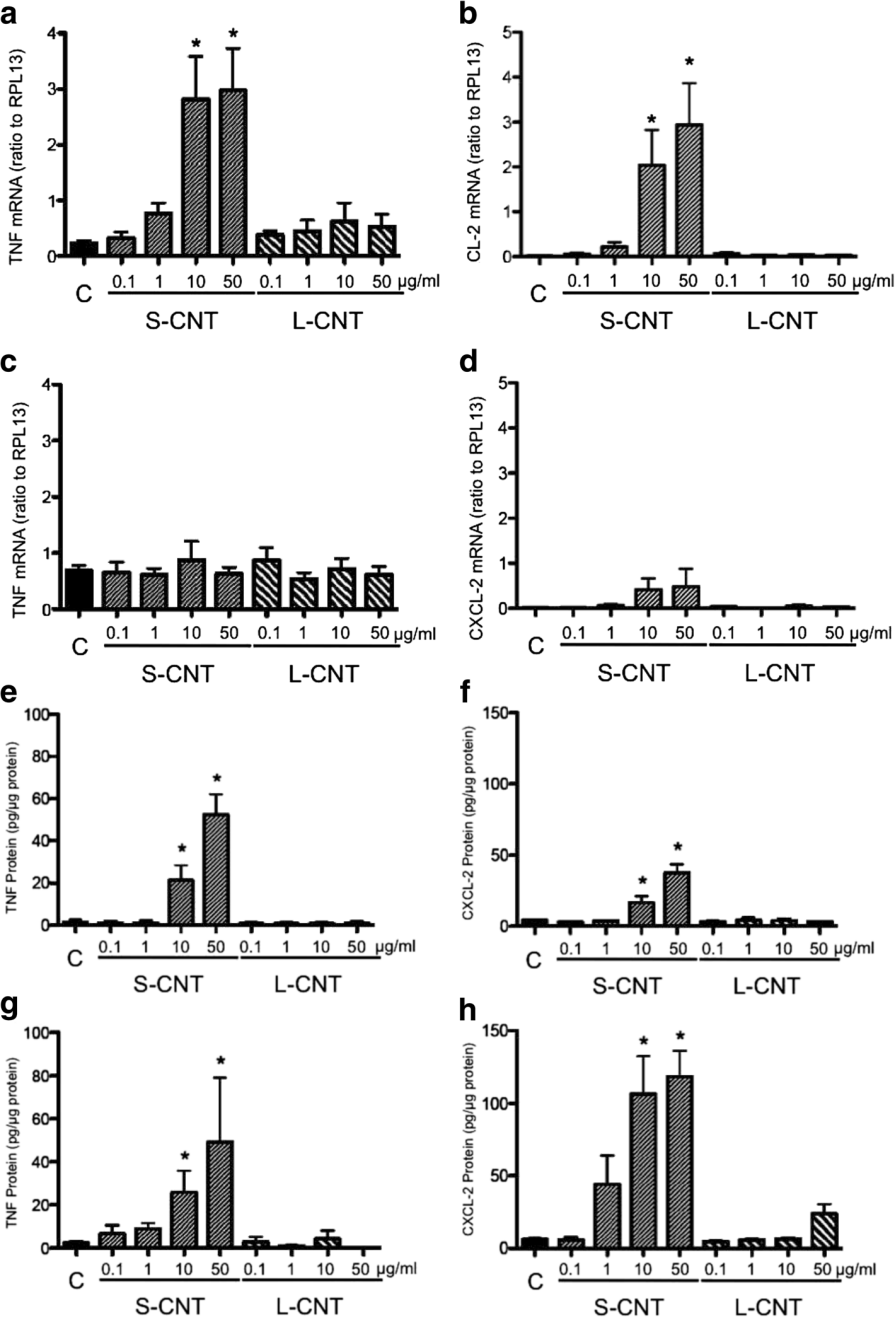
**mRNA and protein expression levels of inflammatory cytokines.** Quantification of mRNA expression levels of TNF (panel **a** and **c**) and CXCL-2 (panel **b** and **d**) in RAW 264.7 macrophages exposed to 0.1-50 μg/mL of S- and L-CNT for 6 (panel **a** and **b**) or 24 (panel **c** and **d**) hours. Quantification of protein expression levels for TNF-α (panel **e** and **g**) and CXCL-2 (panel **f** and **h**) in RAW 264.7 macrophages exposed to 0.1-50 μg/mL of S- and L-CNT for 6 (panel **e** and **f**) or 24 (panel **g** and **h**) hours. *: p<0.05 versus control condition. C: Control (unexposed) cells. S-CNT: short CNT. L-CNT: long CNT.

Expression of the mRNA of both TNF-α and CXCL2 was significantly increased in cells exposed to S-CNT for 6h, but that increase was no longer observable after 24h (Figure 7a to 7d). No significant increase was observed in cells exposed to L-CNT at any time (Figure 7a to 7d). TNF-α and CXCL2 protein levels were increased at both time points in cells exposed to S-CNT but not in those exposed to L-CNT (Figure 7e to 7h). A significant decrease in TNF-α and CXCL-2 protein productions was observed in presence of the antioxidant NAC (Additional file 3: Figure S3a and b), while no modification of those two cytokine levels was observed in presence of the iron chelator Desferrioxamine (Additional file 3: Figure S3c and d).

**Figure 8:**
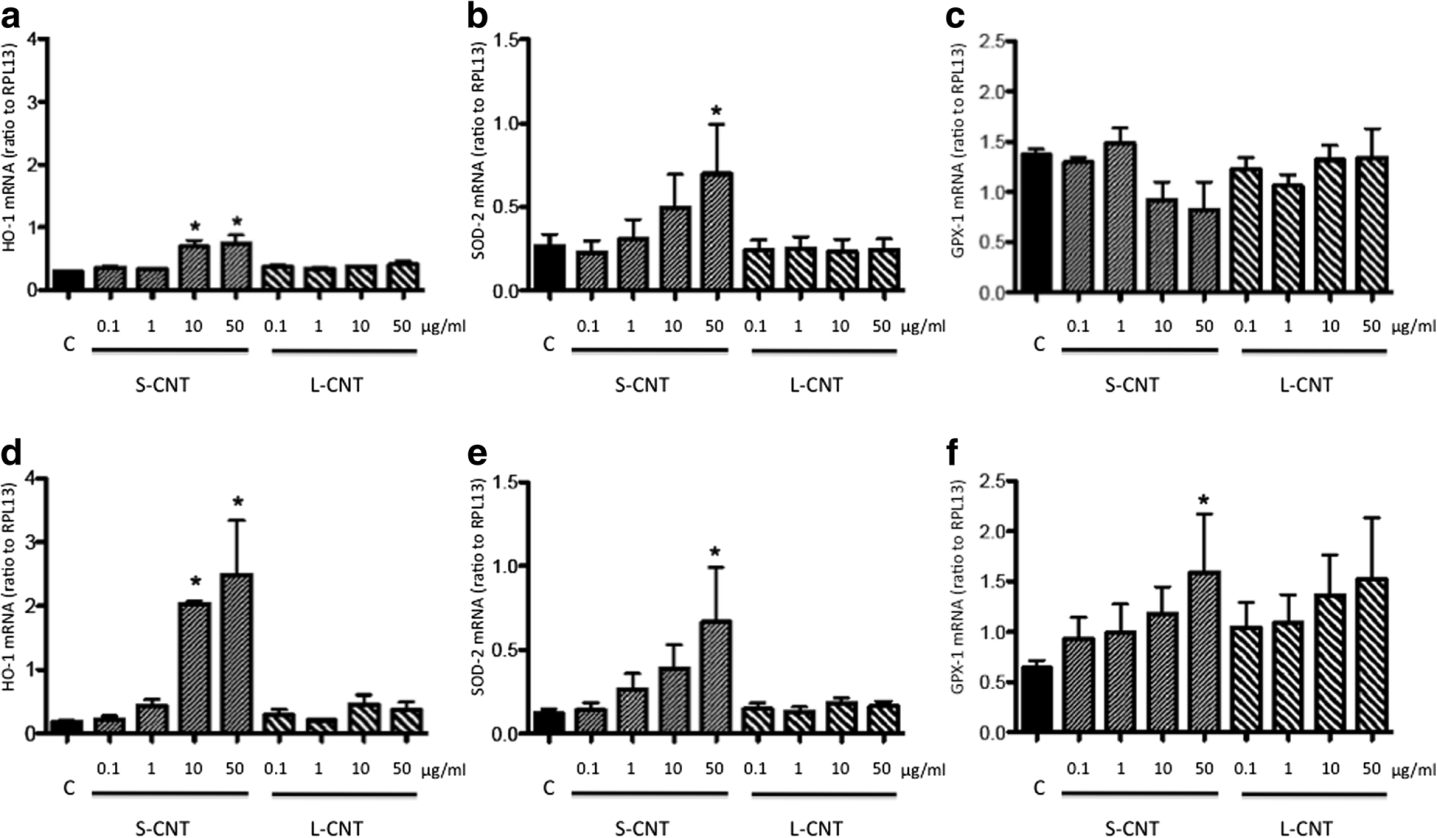
**mRNA expression levels of antioxidant systems.** Quantification of mRNA expression levels for HO-1 (panel **a** and **d**), SOD-2 (panel **b** and **e**) and GPX-1 (panel **c** and **f**) in RAW 264.7 macrophages exposed to 0.1-50 μg/mL of S- and L-CNT for 6 (panel **a**-**c**) or 24 (panel **d**-**f**) hours. *: p<0.05 versus control condition. C: Control (unexposed) cells. S-CNT: short CNT. L-CNT: long CNT.

Expression of the mRNA of two antioxidant genes whose induction is related to cellular oxidative stress, namely heme oxygenase-1 (HO-1) and superoxide dismutase-2 (SOD-2) [31,32] was also measured. Expression levels followed a similar pattern as for pro-inflammatory cytokines: a marked increased expression was observed after 6h of incubation with S-CNT but not when incubating with L-CNT (Figure 8a, b, d, e). This increase was statistically significant after exposure to 10 and 50 μg/ml S-CNT for HO-1, and to 50 μg/ml for SOD-2, at both 6 and 24hr time points. Moreover, the expression of glutathione peroxidase-1 (GPX-1), an enzyme involved in the metabolism of the antioxidant molecule glutathione [33], was only increased 24 hours after the initial exposure of macrophages to 50 μg/ml S-CNT (Figure 8c and f).

## Discussion

Our study was aimed at critically analyzing the toxicity of short and long CNT in murine macrophages. Taken together, our results showed that short and long MWCNT elicited similar reduction in macrophage viability, but only short CNT induced marked dose-dependent pro-inflammatory and pro-oxidative responses. Moreover, thanks to a thorough CNT characterization, our data illustrate how reduction in dimension was inevitably accompanied by variations in other physicochemical characteristics and led us to the conclusion that these additional modifications might be as determinative as the length reduction itself to explain the differences in biological responses between the two samples.

While our aim was to control the physicochemical parameters by using two CNT samples synthesized via the same procedure (iron catalyzed CCVD) so as to control diameter and residual iron catalyst content, the length reduction treatment led to undesired but expected further physicochemical modifications in the shortened CNT sample: *i)* increase in iron oxide nanoparticles residues (two-fold increase of the iron oxide/CNT ratio), *ii)* increase in structural defects (increase of 1% of the sp3/sp2 ratio), together with *iii)* COOH and OH functionalization (4.4 atomic % of increase for surface O amount). Each one or all of these physicochemical modifications could contribute to the higher inflammatory and oxidative response of macrophages to S-CNT compared to L-CNT. First, even though the total iron content was similar in S-CNT and L-CNT (about 5%), XRD analysis showed that the amount of iron oxide (normalized to that of CNT) was two times higher in the former than in the latter CNT sample. Since iron nanoparticles were mainly localized inside the hollow core of the CNT, their oxidation in the S-CNT sample suggests that they were in contact with water during the shortening treatment, probably as a consequence of a breaking/tip opening process. More important, this also suggests that iron nanoparticles in S-CNT sample may have been later in contact with the biological medium, during cell exposure. Some published data indicate that the presence of bio-available metallic elements, such as iron, plays a critical role in CNT toxicity, through the induction of an intracellular oxidative stress [34,35]. It is indeed well-known that transition metals may contribute to particle-induced reactive oxygen species (ROS) generation through mechanisms such as a Fenton reaction, leading, together with cell-derived ROS, to oxidative stress [36]. The fact that two markers of oxidative stress were induced exclusively in the macrophages exposed to S-CNT, together with the protective effect of NAC on pro-inflammatory cytokine production, supports the idea that the induction of an oxidative stress was involved in the development of the inflammatory response observed in S-CNT exposed cells, and that the suspected bioavailable iron in S-CNT may be ascribed for the oxidative stress induction. In order to address the question whether iron from the residual catalyst particles was responsible for the oxidative and inflammatory effects of S-CNT, cells were incubated with the iron chelator desferrioxamine. These experiments did not provide any evidence for the iron contained in S-CNT to take part in the CNT-induced effects. Therefore, other modifications brought by the shortening process should be responsible for the specific induction of oxidative stress and inflammation with S-CNT.

The second major modification introduced by CNT shortening was the presence of structural defects. Fenoglio and collaborators [37] and Muller and coworkers [38] have demonstrated that MWCNT presenting a defective carbon framework induced higher inflammatory and genotoxic responses than MWCNT without these defects. The authors related these effects to the capacity of CNT to scavenge ROS; more defects were associated to an increased scavenging activity. We did not analyze ROS scavenging capacity of S-CNT, but if this capacity was present, one could expect an absence of oxidative stress, as shown with fullerenol C_60_(OH)_22_[39]. Since exposure to S-CNT was associated with the expression of oxidative stress markers (HO-1 and SOD-2 mRNA), a ROS scavenging capacity of S-CNT consecutive to the presence of structural defects is unlikely. The difference in structural defects between the 2 CNT samples can therefore be ruled out to explain the difference in biological response.

Finally, the 3^rd^ modification that was introduced in S-CNT and could be related to their inflammatory and oxidative effects is the presence of functional groups at their surface. Tabet and collaborators, using an approach consisting in embedding MWCNT with an acidic polymer, showed that acidic polymer-embedded MWCNT induced a higher inflammatory response (total cell number in broncho-alveolar lavage (BAL) fluid, production of TNF and CXCL-2) than pristine MWCNT or hydrophobic polymer-embedded ones [14]. This was also associated with a higher cellular uptake of acidic polymer-embedded MWCNT by BAL macrophages, and the authors suggested that the COOH groups might play a role in the inflammation induced by such coated MWCNT. Similarly, Saxena and collaborators [40] showed that acid-functionalized SWCNT were more potent than pristine-SWCNT in inducing mouse lung epithelial cell cycle arrest and lung inflammation. They related these effects to a better dispersion resulting from their negative charges and leading to a high bioavailability of these CNT and/or to their negative charges. The same group of investigators demonstrated that acid-functionalization also enhanced cardiac toxicity of SWCNT after pulmonary exposure [41]. Although these last two studies did not examine the effect of acid-functionalization on SWCNT cellular uptake or oxidative stress, they support the hypothesis of a role of COOH groups in the inflammatory and oxidative responses induced by the S-CNT. The absence of inflammatory and oxidative responses with the L-CNT without COOH groups agree also well with this hypothesis, and stress the paramount importance of surface properties/chemistry in determining biological impact of CNT.

In the present work, the number of cells internalizing CNT in vesicles was higher for S-CNT-exposed cells compared to L-CNT-exposed ones, with a similar number of CNT incorporated in each cell. As the initial aim of our study was to evaluate the role of CNT length in their cytotoxicity, we chose to prepare our samples without any additive to the culture medium. This led to sedimentation and the formation of aggregates for both CNT samples. As observed in the optical microscopy images, and thanks to observations at the early stages of the exposures, we couldn’t observe any obvious difference, which could have modified their internalization, between the 2 samples in the aggregation pattern or the way CNT sediment on cells over time. For both CNT, the length of internalized CNT was largely smaller than 15 μm, which is the currently proposed cut-off size over which CNT can induce an inflammatory response [21,23]. These results are in accordance with data showing that CNT with length superior to 10 μm are poorly internalized [42]. Interestingly, although the mean length of internalized S-CNT was higher than that of L-CNT, the length distribution of internalized CNT was quite large, with overlapping values for S-CNT (0.14 to 2.65 μm) and L-CNT (0.13 to 1.42 μm). Beside length, the physicochemical determinants ruling CNT cellular uptake are still poorly known. However, surface properties have been suspected to play a major role in the CNT-cell interaction and further internalization. Kostarelos and coworkers examined the uptake of a wide variety of functionalized CNT by different cell types [43] and concluded, as all CNT were internalized, that the nature of the functional groups on the CNT surface did not determine whether the CNT were internalized. However, these authors did not examine CNT displaying characteristics similar to those of the S-CNT studied in our work. In agreement with our findings, Tabet and coworkers [14] showed that variation in the nature of the polymer used to coat MWCNT was associated with differential internalization of CNT inside macrophages; hydrophilic acidic polymer-coated MWCNT were significantly more internalized than the hydrophobic polymer-coated ones. The penetration of CNT through the plasma membrane in a “nano-syringe”-like fashion has been theoretically demonstrated [44], and molecular dynamics simulations have confirmed that this phenomenon could be related to hydrophilic functionalities present on the surface of oxidized materials which spontaneously insert inside the cell membrane by a lipid-assisted mechanism [44,45]. In our study, similar nano-syringe phenomena were also observed by TEM for S-CNT-exposed cells (Additional file 1: Figure S1b). Given that S-CNT present carboxylic and hydroxyl groups on their surface, such kind of phenomenon (lipid-assisted mechanism mediated by hydrophilic functionalities) could therefore explain the higher cellular incorporation of S-CNT.

No direct relationship between CNT internalization and inflammatory response has been clearly established yet [14,46-48], but it would be tempting to explain these singular responses to S-CNT by the higher cellular uptake observed for these CNT. In order to evaluate this hypothesis, we took into account the percentage of CNT-positive cells (i.e. cells containing CNT) when interpreting the results of our cytokine and oxidative stress assays. The percentage of cells containing CNT within vesicles was 2.35 higher with S-CNT than with L-CNT (25.33% versus 10.77% for S-CNT and L-CNT respectively), while the number of CNT contained inside each vesicle was similar for both CNT. At the same time, TNF-α and CXCL-2 secretions were respectively 6 times and almost 17 times higher in response to S-CNT exposure than in response to L-CNT exposure (25.78 versus 4.31 ng/ml for TNF-α and 106.48 versus 6.38 ng/ml for CXCL-2, respectively). Therefore, the increase in inflammatory response (revealed by the amount of cytokines produced by cells exposed to S-CNT compared to L-CNT) was clearly more important than the difference in CNT content per cell between the two groups. Interestingly, a similar pattern was observed for HO-1 and SOD-2 mRNA expression. Taken together, these results strongly suggest that the enhanced inflammatory and oxidative responses to S-CNT were not only a consequence of a higher uptake of S-CNT by cells, but could also result from the material's intrinsic characteristics (length, surface features) that varied between S-CNT and L-CNT. Both S- and L-CNT induced a similar decrease in cellular viability, but, as discussed earlier, only exposure to S-CNT was associated with increased pro-inflammatory and pro-oxidative responses. Similar dissociation between cell mortality and inflammatory and/or oxidative response has been described in the literature [14,49-51]. In the present case, the difference observed between the two CNT could be due to different cellular pathways targeted by S- and L-CNT, such as what has been described for MWCNT and asbestos in lung epithelial cells [51], or MWCNT embedded in different polymers [14]. Another possibility could be a preferred interaction of proteins and/or DNA with L-CNT compared to S-CNT, further leading to false negative results when the amount of inflammatory proteins or DNA was quantified after cell incubation with L-CNT. However, internal controls in the experimental set-up allow us to rule out such possibility.

The absence of an inflammatory effect of L-CNT is all the more surprising since these CNT have a similar length distribution to those eliciting a clear inflammatory response (both *in vitro* and *in vivo*) in studies by Donaldson and coworkers (i.e. CNT_long1_) [21,23]. In those studies, the inflammatory reaction induced by long CNT has been related to a phenomenon called “frustrated phagocytosis”, which is characterized by macrophages not being able to eliminate long and rigid fibers because of incomplete engulfment [52]. Experimental studies suggest that “frustrated phagocytosis” has a dramatic influence on the sustained generation of ROS [53], which in turn contribute to the secretion of inflammatory mediators [54,55]. In our study, almost no frustrated phagocytosis was observed either for S-CNT or L-CNT. Given the complexity of *in vivo* environment, care should be taken when comparing *in vitro* and *in vivo* data. Indeed, frustrated phagocytosis is a critical issue for fiber toxicity, but an equivalent important issue is particle clearance [22,56] that can hardly be evaluated by *in vitro* studies. To explain the difference between our findings with L-CNT and the results from Donaldson and coworkers with long CNT, one hypothesis is the use in our experiments of less rigid CNT that lead to less frustrated phagocytosis than rigid ones [16,57]. We did not measure the rigidity of L-CNT, but our CNT were thinner than the ones used by Donaldson and coworkers (mean diameter 42 vs 85 nm respectively) [21] suggesting that they could be bent more easily, and therefore be less subjected to frustrated phagocytosis and more fully engulfed.

## Conclusion

In conclusion, our results stress the difficulty to address the role of one single physico-chemical parameter at a time when dealing with CNT biological effects, even though a controlled synthesis procedure was used. Surface properties should be considered as essential determinants, alongside the length, in CNT-induced oxidative stress and inflammation, especially when dealing with the safe design of CNT for applications in nanomedicine.

## Methods

### CNT production

Two MWCNT samples (S-CNT precursor and L-CNT) were produced by aerosol-assisted CCVD. The method is based on the catalytic decomposition of liquid hydrocarbons by pyrolysing mixed aerosols containing both the hydrocarbon and the metallic source which simultaneously and continuously fill the reactor [25]. A solution composed of ferrocene dissolved in toluene (2.5 wt.% for PS-CNT and 5 wt.% for L-CNT) was used to synthesize the two CNT samples at 850°C. Following this procedure, samples are formed of aligned CNT carpets covering the reactor walls. The duration of the aligned growth of CNT was fixed at 10min for PS-CNT and only 2min for L-CNT. Once detached from the reactor walls by scrapping off, PS-CNT sample was treated in de-ionized water for 7 weeks using ultrasonic bath (25 kHz, 100% power) in order to shorten the CNT and reach a desired length distribution. The final dry sample of S-CNT was obtained by evaporating water in a fume hood.

### CNT characterization

#### Optical, scanning electron and transmission electron microscopies

Samples were observed using optical (Olympus BX 60 optical microscope coupled to a Color view digital camera), scanning electron (SEM, FEG-SEM; Carl Zeiss Ultra 55, field emission gun) and transmission electron (TEM) microscopies to evaluate the quality of the MWCNT (i.e. morphology, structure, and presence of synthesis by-products), and also to determine the length distribution. Morphology and thickness of the CNT carpets were investigated by SEM on cross sections of aligned CNT carpet grown on reference quartz substrate (PS- and L-CNT) which were fixed on the SEM sample holder with a carbon adhesive tape. Beam voltage was 5 kV, working distance 3 mm, and size aperture 30μm. We used SE2 or InLens electron detectors. To perform TEM analysis, CNT powder was dispersed in ethanol with US bath for less than 1 min. One droplet of this suspension was then deposited on a Cu grid covered with lacey carbon film. Grids were observed on a Philips CM12 TEM microscope operating at 120kV.

#### Thermogravimetric analysis

Thermogravimetric analysis (TGA 92–16, 18 SETARAM apparatus) was performed under flowing air at a temperature up to 1000°C (10°C min^-1^ heating ramp) to determine the sample initial iron content by measuring the remaining iron oxide weight.

#### X-ray diffraction

X-ray diffraction (XRD) experiments were carried out in transmission geometry on a rotating anode generator. The Molybdenum Kα X-ray radiation was used as incident wavelength (λ = 0.711 Å) so that fluorescence from iron-based particles was relatively low. Collimator, sample, and detector were altogether placed in a vacuum chamber in order to minimize air scattering. Dry samples (i.e. MWCNT powders) were placed into glass capillaries. A two-dimensional phosphorescent imaging plate was used as the detector; the signal was then integrated angularly to obtain the wave-vector dependence of the scattered intensity.

#### X-ray induced photoelectron Spectroscopy

The surface chemical composition of both S-CNT and L-CNT samples was determined by XPS (X-ray induced Photoelectrons Spectroscopy) using a Kratos Analytical Axis Ultra DLD spectrometer with monochromatic Al Kα X-ray radiation (hν = 1486.6eV). C1s, O1s and Fe2p spectra were recorded at a take-off angle of 90° with a 700μm by 300μm slot aperture and 20eV pass energy. The energy scale of the instrument was calibrated by setting Au 4f7/2 = 84.0 eV, Ag3d5/2 = 368.7 eV. Data from 3 independent measures were acquired with Kratos Analytical Vision 2 software. Peak fitting was performed after Shirley baseline background subtraction [58] using Thermo Electron Software. A Lorentzian/Gaussian ratio of 70% was applied to sp2 carbon peak and 30% to other C1s, O1s, Si2p and Ti2p oxide peaks. The energy of sp3 carbon peak was fixed to 285.1 eV with a full width at half maximum (FWHM) of 1.5 eV. The atomic sensitivity factors used for semi-quantitative analysis were those given by Scofield [59] (C1s = 1.0, O1s = 2.93, Fe2p3/2 = 10.82 and Si2p =0.82, relative to C1s = 1.00).

### Endotoxin contamination of CNT

S- and L-CNT samples were assessed for endotoxin contamination using the Limulus Amebocyte Lysate assay (Lonza), performed as per the manufacturer's instructions.

### Cell culture and exposure to CNT

RAW 264.7 murine macrophages were purchased from the American Type Culture Collection (Manassas, VA). Cells were cultured in Dulbecco's Modified Eagle Medium (DMEM) supplemented with 10% heat-inactivated fetal calf serum and antibiotics (streptomycin, 10 mg/mL; penicillin G, 10000 IU/mL; and amphotericin B, 25 μg/mL) at 37°C in a humidified atmosphere of 5% CO_2_/95% air. Sub-confluent cells were then exposed for 6 or 24 hours to a scaling dose of MWCNTs (0.1-50 μg/ml; 0.2-20 μg/cm^2^) prepared by dispersion of the dry material sample in serum-free cell culture medium. For homogenization purpose, the MWCNT suspension was US bath-sonicated and vortexed just before cell stimulation. In a subset of experiments, cells were pretreated with the antioxidant N-Acetyl Cystein (NAC, 2 mM) 1 hour prior to CNT exposure, or with the iron chelator Desferrioxamine (DEF, 100 μM, as previously described [60]).

### Morphology of cells exposed to CNT

Cell morphology was evaluated by optical microscopy after standard Harris haematoxylin-eosin/phloxin staining of cells exposed for 24h to 10μg/mL of CNTs.

### Cellular uptake of CNT

Cells exposed for 24 h to 10 μg/ml of CNTs were analyzed by TEM as described previously [61]. Briefly, cell monolayers were resin-embedded and then processed as to prepare semi-thin sections (2 by 2mm; 200nm thick) for ultra-structural cytology and ultra-thin sections (0.5 by0.5mm; 70nm thick) for TEM analysis.

The percentage of cells having internalized CNTs in vesicles was analyzed by optical microscopy on Toluidine blue-stained semi-thin sections of the cell monolayer (2 by2mm, prepared from TEM block specimen). For each condition of stimulation, 5 fields were selected from the top to the bottom across the semi-thin section. Analysis was performed blinded by 2 independent observers (SL and CB). The coefficient of variation for the measurement was <5%.

The calculation of the number and length of internalized CNT was performed on ultra-thin TEM sections. For each condition of stimulation, 15 fields were selected from the top to the bottom across the ultra-thin section, and a minimum of 50 cells per sample was observed. CNT number was evaluated in vesicles only (since CNT in cytoplasm are very difficult to observe), and CNT length was measured both in vesicles and free in the cytoplasm. Analysis was performed blinded by 3 independent observers (CB, MP and SL). The coefficient of variation for the measurement was <5%.

### Cell viability

Cellular viability was assessed using 2 methods: WST-1 assay, and the quantification of DNA content. These tests were performed as previously described [51]. Results were expressed as the means of at least 3 independent experiments, each of 6 replicates, given as the ratio of the mean for each condition to the mean of the control condition (cells exposed to DMEM). Since nanomaterials could interfere with cytotoxicity tests [62,63], we performed the assays incubating dyes with nanotubes only (100 μg/ml of S-CNT or L-CNT) and then measured absorbance. No positive or negative interference of S-CNT or L-CNT with any assays was observed (data not shown).

### Reverse transcription and quantitative PCR (Q-PCR)

Quantification of the mRNA expression of different genes involved in oxidative stress and inflammation was performed by quantitative RT-PCR as described previously [51]. Primer sets are shown in Table 2. The expression of the gene of interest was reported as the ratio to the housekeeping RPL13 gene expression. To evaluate a possible interference of CNT with the different steps of the Q-PCR experiment (mRNA isolation, reverse transcription and polymerase chain reaction), each step was performed in Control samples, in presence or in absence of 100 μg/ml CNT. No modification of the efficiency of each step was observed (data not shown).

**Table 2:** Primers used for real-time quantitative PCR

**Gene**	**Forward primer**	**Reverse primer**
*RPL-13*	GTGGTCCCTGCTGCTCTCCAA	CGATAGTGCATCTTGGCCTTTT
*HO-1*	CACGCATATACCCGCTACCT	CCAGAGTGTTCATTCGAGCA
*GPX-1*	TGAAGAGATTCTGAATTCCCTCAAG	CAGGAAGGTAAAGAGCGGGTG
*SOD-1*	CAAATTACAGGATTAACTGAAGGCC	GGCCACCATGTTTCTTAGAGTGAG
*TNF-α*	CTGTCTACTGAACTTCGGGGTGAT	GGTCTGGGCCATAGAACTGATG
*CXCL2*	GAACATCCAGAGCTTGAGTGTGAC	CTTGCCTTTGTTCAGTATCTTTTGG

*RPL13*, ribosomal protein L13; *HO-1*, heme oxygenase-1; *SOD*, superoxide dismutase; *GPX-1*, Glutathione peroxidase-1; *CXCL2*, macrophage inflammatory protein-2; *TNF-α*, tumour necrosis factor alpha.

### ELISA

The concentration of the proinflammatory cytokine TNF-α and the chemoattractant chemokine CXCL2 in culture supernatant was determined by ELISA (R&D Systems, Lille, France), as previously described [60]. Interference of NP with ELISA assay was assessed by quantifying the amount of known concentrations of TNF-α or CXCL2 in presence or in absence of CNT. No interference was observed (Additional file 4: Figure S4). Results are expressed as pg/μg protein.

### Statistical analysis

Each value is the mean ± Standard Error of the Mean (SEM) of at least 4 experiments performed in triplicate. Data were analyzed with the GraphPad Prism 4.0 software (La Jolla, CA, USA). Comparisons between multiple groups were performed by using Kruskall–Wallis’ non-parametric analysis of variance test followed, when a difference was detected, by two-by-two comparisons with the Mann–Whitney’s U test. P-values <0.05 were considered significant.

## Competing interests

Authors have no competing interests to declare.

## Authors’ contribution

CB, PL, JB and SL participated in the design the study. CB performed all biological experiments, and participated in the physico-chemical characterization of CNT. MP and MML synthesized and characterized the CNT. JC, PJ and PL performed XRD and XPS analysis and analyzed the characterization data. MJL performed cytokine assays. JB and SL wrote the first draft of the manuscript. All authors read and approved the final manuscript.

## Supplementary Material

Additional file 1 **Figure S1.** Transmission electron microscopy images of S- and L-CNT. Transmission electron microscopy (TEM) images of S-CNT and L-CNT, free in the cytoplasm (panel a). Panel b shows representative image of S-CNT penetrating through the plasma membrane. Click here for file

Additional file 2 **Figure S2. **Viability of macrophages exposed to S- and L-CNT. Quantification of cell viability using MTT assay in RAW 264.7 macrophages exposed to 0.1-50 μg/mL of S- or L-CNT for 6 (panel a) or 24 (panel b) hours. *: p<0.05 versus control condition. S-CNT: short CNT. L-CNT: long CNT. Click here for file

Additional file 3 **Figure S3.** Protein expression levels of inflammatory cytokines in presence of DEF. Quantification of protein expression levels for TNF-α (panel a and c) and CXCL-2 (panel b and d) in RAW 264.7 macrophages exposed to 50 μg/mL of S- and L-CNT for 24 hours, in presence or absence of 2 mM NAC (panel a and b) or 100 μM DEF (panel c and d). *: p<0.05 versus control condition. #: p<0.05 vs S-CNT without NAC. C: Control (unexposed) cells. S-CNT: short CNT. L-CNT: long CNT. NAC: N-Acetyl Cystein. DEF: Desferrioxamine. Click here for file

Additional file 4 **Figure S4.** Protein expression of TNF-α in presence or absence of S- or L-CNT. Quantification of TNF-α protein expression by ELISA. Two known concentrations of TNF-α (21.9 and 350 pg/ml respectively) were incubated in presence of in absence of 50 μg/ml S- or L-CNT to assess for interference between CNT and proteins. Black bars are for TNF-α alone. Dashed bars are for S-CNT. Anti-dashed bars are for L-CNT. Click here for file
