# Supplementary figures and images for: Critical role of surface chemical modifications induced by length shortening on multi-walled carbon nanotubes-induced toxicity
Source: Part Fibre Toxicol. 2012 Nov 27;9:46. doi: 10.1186/1743-8977-9-46 (PMC3515433; doi:10.1186/1743-8977-9-46)

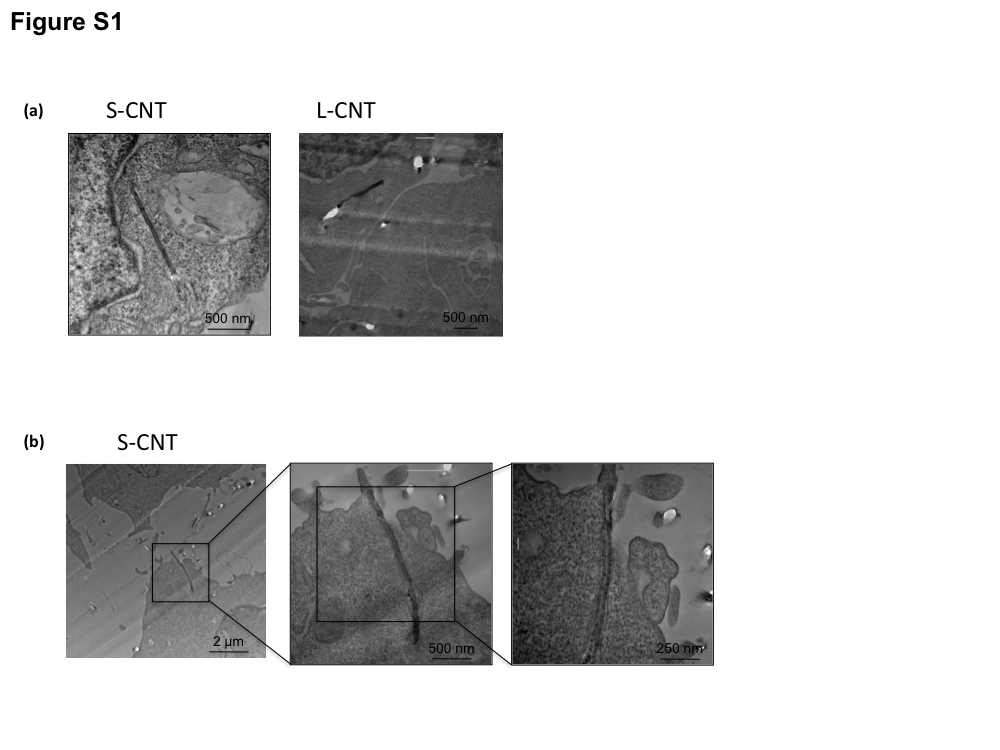

Supplement: Additional file 1 — Figure S1. Transmission electron microscopy images of S- and L-CNT. Transmission electron microscopy (TEM) images of S-CNT and L-CNT, free in the cytoplasm (panel a). Panel b shows representative image of S-CNT penetrating through the plasma membrane. [file 1743-8977-9-46-S1.tiff]

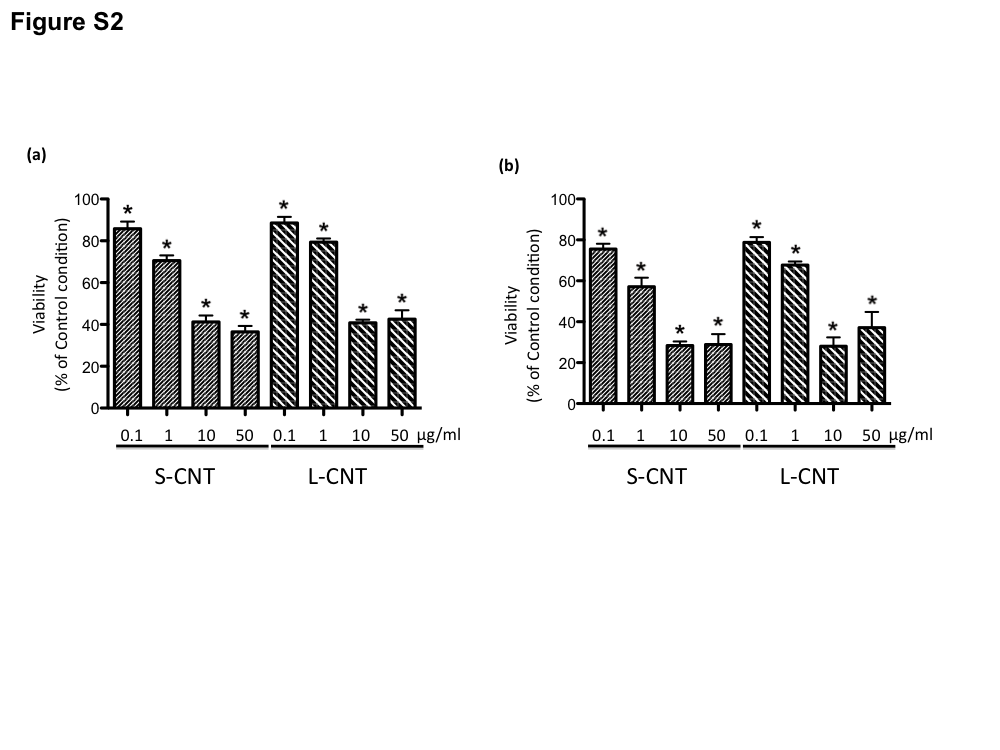

Supplement: Additional file 2 — Figure S2. Viability of macrophages exposed to S- and L-CNT. Quantification of cell viability using MTT assay in RAW 264.7 macrophages exposed to 0.1-50 μg/mL of S- or L-CNT for 6 (panel a) or 24 (panel b) hours. *: p<0.05 versus control condition. S-CNT: short CNT. L-CNT: long CNT. [file 1743-8977-9-46-S2.tiff]

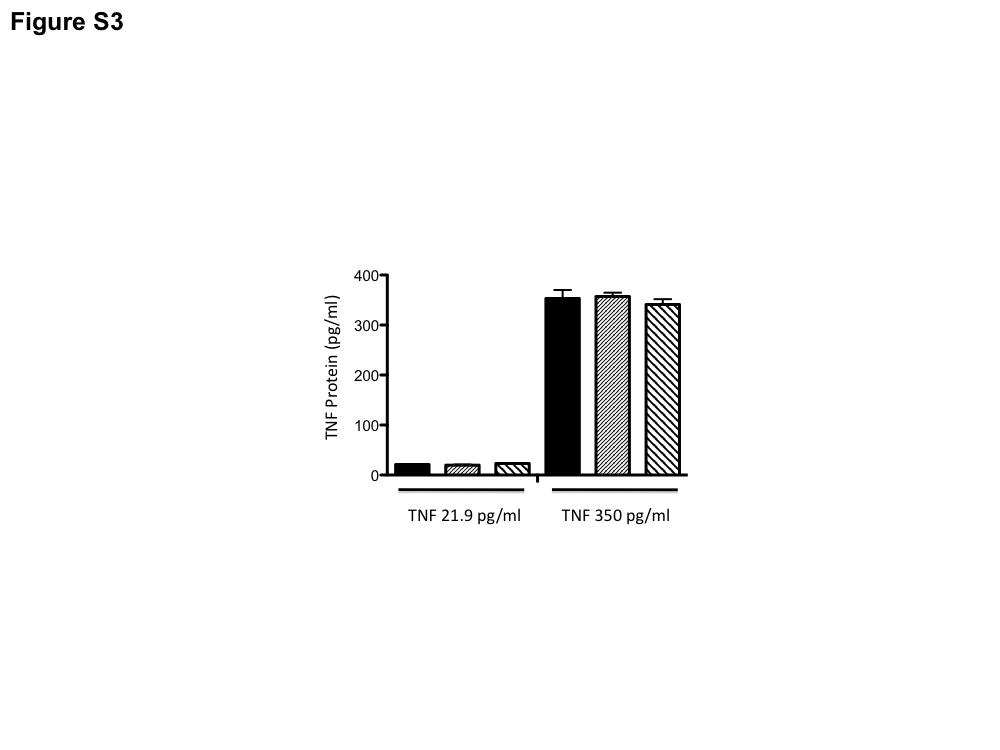

Supplement: Additional file 3 — Figure S3. Protein expression levels of inflammatory cytokines in presence of DEF. Quantification of protein expression levels for TNF-α (panel a and c) and CXCL-2 (panel b and d) in RAW 264.7 macrophages exposed to 50 μg/mL of S- and L-CNT for 24 hours, in presence or absence of 2 mM NAC (panel a and b) or 100 μM DEF (panel c and d). *: p<0.05 versus control condition. #: p<0.05 vs S-CNT without NAC. C: Control (unexposed) cells. S-CNT: short CNT. L-CNT: long CNT. NAC: N-Acetyl Cystein. DEF: Desferrioxamine. [file 1743-8977-9-46-S3.tiff]

**Figure S4**

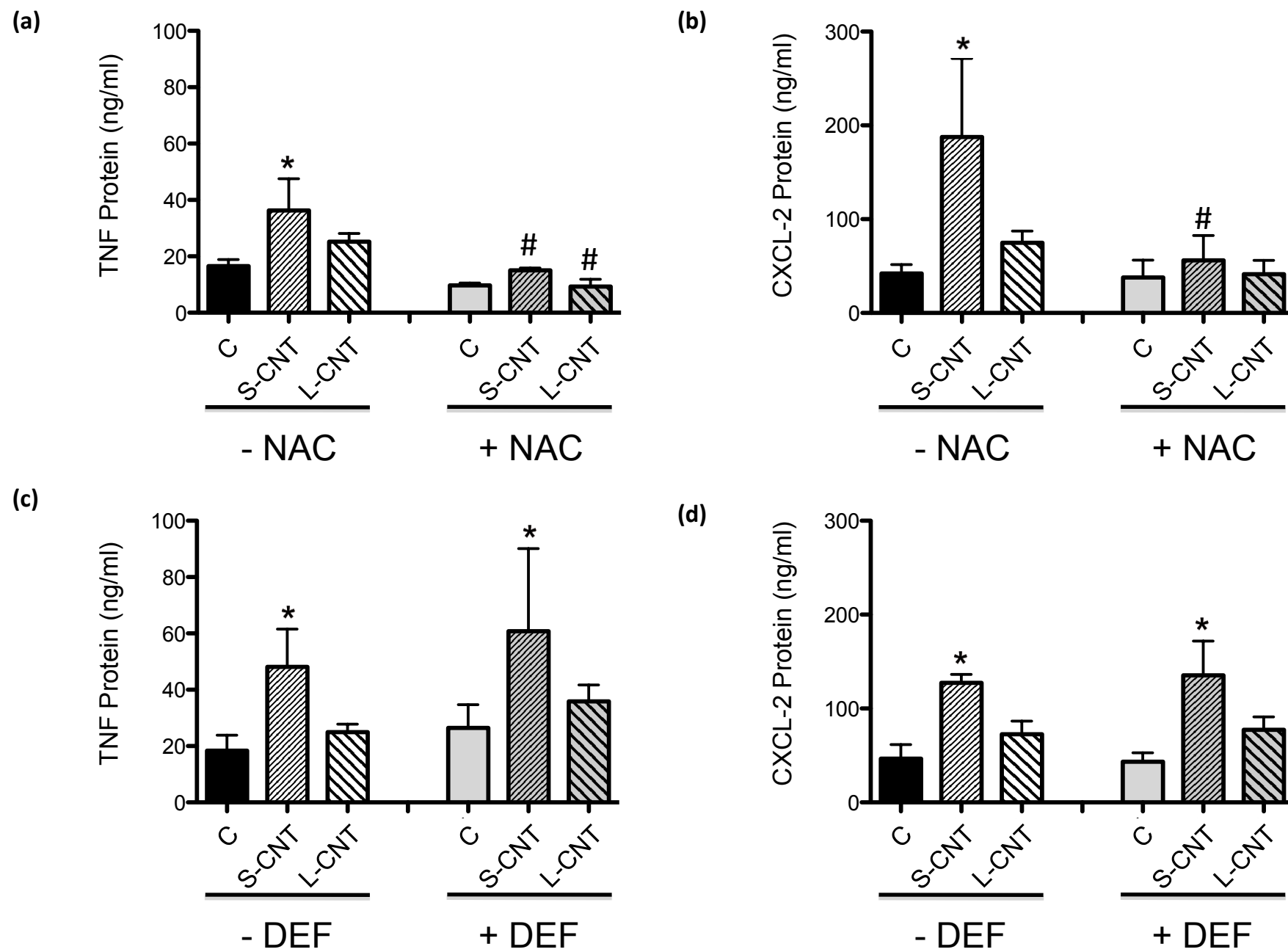

Supplement: Additional file 4 — Figure S4. Protein expression of TNF-α in presence or absence of S- or L-CNT. Quantification of TNF-α protein expression by ELISA. Two known concentrations of TNF-α (21.9 and 350 pg/ml respectively) were incubated in presence of in absence of 50 μg/ml S- or L-CNT to assess for interference between CNT and proteins. Black bars are for TNF-α alone. Dashed bars are for S-CNT. Anti-dashed bars are for L-CNT. [file 1743-8977-9-46-S4.pdf]
